# Comparative effects of various types of toric intraocular lenses on astigmatism correction

**DOI:** 10.1186/s12886-020-01439-4

**Published:** 2020-04-28

**Authors:** Jayoon Moon, Chang Ho Yoon, Mee Kum Kim

**Affiliations:** 1 Department of Ophthalmology, Seoul National University Hospital, 101 Daehak-ro, Jongno-gu, Seoul, 03080 Korea; 2 Department of Ophthalmology, Seoul National University College of Medicine, 103 Daehak-ro, Jongno-gu, Seoul, 03080 Korea; 3 Laboratory of Ocular Regenerative Medicine and Immunology, Biomedical Research Institute, Seoul National University Hospital, 103 Daehak-ro, Jongno-gu, Seoul, 03080 Korea

**Keywords:** Astigmatism, Cataract, Toric intraocular lens, Front-toric, Back-toric, Bi-toric

## Abstract

**Background:**

Currently, various types of toric intraocular lenses (IOL) have been manufactured and can be divided into three types according to the location of correction component; front-toric IOL (correction on anterior IOL surface), back-toric IOL (correction on posterior IOL surface), and bi-toric IOL (correction on both anterior and posterior IOL surfaces). In this study, we aimed to investigate the effectiveness of reducing corneal astigmatism of either normal or post-penetrating keratoplasty (PKP) corneas according to the type of implanted toric IOLs.

**Methods:**

Medical records were retrospectively reviewed in 370 patients who had undergone phacoemulsification with posterior chamber toric IOL insertion (front-toric IOL, back-toric IOL or bi-toric IOL). Subjects were divided into 2 groups; subjects who had no history of corneal disease with corneal astigmatism more than 1.00 diopters (D) (G1) and subjects who received previous PKP with all corneal sutures removed and had corneal astigmatism more than 1.25 D (G2). Preoperatively intended target from SRK/T was evaluated. Refractive astigmatism and its vector analysis (J0, J45), mean numerical error (MNE) and mean absolute error (MAE) were assessed at least a month after cataract surgery.

**Results:**

Mean preoperative corneal astigmatisms were 2.2 D and 4.0 D in G1 and G2, respectively. There was significant reduction of mean postoperative refractive astigmatism to 0.89 D in G1 and to 2.33 D in G2. In G1, bi-toric IOL showed significantly more improved refractive astigmatism than back-toric IOL. In G2, no difference in refractive astigmatism according to toric IOL type was observed. While G2 showed no difference in MNE among toric IOLs, in G1, bi-toric IOL showed significant hyperopic shift compared to back-toric IOL. In both groups, there was no significant difference in MAE according to type of IOL. No postoperative complications were observed.

**Conclusion:**

Our study suggests that all types of toric IOL are beneficial in correcting astigmatism of normal and post-PKP corneas. Noticeably, bi-toric IOL showed significantly better results in refractive astigmatism than back-toric IOL in normal cornea. However, bi-toric IOL showed a more hyperopic shift compared to back-toric IOL. Among post-PKP corneas, all types of toric IOL showed similar results.

## Background

The importance of astigmatism’s influence in vision is increasing along with the advancement of technology and surgical procedures to correct corneal astigmatism during cataract surgery. About 20% of all patients undergoing cataract surgery are known to have preoperative corneal astigmatism over 1.50 diopters (D) [1]. Therefore, visual improvement is insufficient solely with spherical intraocular lens (IOL) during cataract surgery. Many studies have proven visual enhancement through several corneal refractive procedures in addition to cataract surgery [2, 3]. Recent studies have proven that implantation of toric IOLs are an effective noninvasive procedure in correcting corneal astigmatism [4–7]. Also, several studies show that toric IOLs are more beneficial in correcting corneal astigmatism compared to non-toric spherical IOL combined with limbal relaxing incisions [4, 6].

High corneal astigmatism is known to be one of the most common causes for insufficient visual performance with low patient satisfaction after penetrating keratoplasty (PKP). Average corneal astigmatism after PKP is known to be as high as 6 D [8]. To date, several studies have proven that implantation of toric IOLs significantly reduces post-PKP corneal astigmatism and improves visual acuity [9–12].

Currently, various types of toric IOLs have been manufactured and can be divided into three types according to the location of correction component; front-toric IOL (correction on anterior IOL surface), back-toric IOL (correction on posterior IOL surface), and bi-toric IOL (correction on both anterior and posterior IOL surfaces). Several comparative studies have been conducted and have proven similar visual outcomes and reduction of overall astigmatism without significant differences among customized toric IOLs [12–15]. According to the manufacturer, bi-toric IOL can reduce very high astigmatism by providing cylinder power up to + 12.00 D. Therefore, the effects of astigmatism reduction may be different between moderate astigmatism in normal corneas and high astigmatism in post-PKP corneas depending on IOL types.

In this study, we aimed to investigate the effectiveness of reducing corneal astigmatism of either normal or post-PKP corneas according to the type of implanted toric IOLs.

## Methods

### Subjects and types of IOL

This study was approved by the Institutional Review Board of Seoul National University Hospital (IRB No. 1903–144-1020, Seoul, South Korea) and was conducted with adherence to Declaration of Helsinki. Medical records of patients who had received uneventful phacoemulsification with toric IOL implantation between January 1st, 2010 and August 31st, 2017 at Seoul National University Hospital (Seoul, Korea) were retrospectively reviewed. The surgery was performed by one surgeon (M.K. Kim). Subjects were divided into 2 groups (*n* = 370 eyes); subjects who had corneal astigmatism of 1.00 D or more without prior or present corneal diseases were included in group 1 (G1, *n* = 349 eyes) and subjects who had received PKP and full corneal suture removal at least 6 months prior to cataract surgery with corneal astigmatism 1.25 D or more and a clear graft were included in group 2 (G2, *n* = 21 eyes). Exclusion criteria were as follows; subjects who were younger than 18 years of age, had present ocular diseases in cornea, vitreous, retina or optic nerves that may limit visual acuity and had incomplete or poor clinical data.

Three types of toric IOL were used in this study; Acrysof toric (Alcon Laboratories, Inc., back-toric IOL, *n* = 174 eyes in G1, *n* = 13 eyes in G2), Tecnis toric (Abbott Medical Optics, Inc., front-toric IOL, *n* = 85 eyes in G1, *n* = 2 eyes in G2), and Zeiss toric (AT TORBI 709 M, Carl Zeiss Meditec AG, bi-toric IOL, *n* = 90 eyes in G1, *n* = 6 eyes in G2).

### Study design

In G1 and G2, visual acuity (logMAR), minus cylinder powers of refractive and corneal astigmatisms and their vector analysis, and difference between preoperatively intended spherical equivalents (SE) and final postoperative SE were compared depending on type of toric IOLs.

All subjects had undergone preoperative and postoperative assessments of uncorrected distance visual acuity (UCVA) and best corrected distance visual acuity (BCVA), keratometry measurements by autokeratometer (KR-8100, Topcon, Tokyo, Japan), examination with IOL master 500 (Carl Zeiss Meditec AG, Jena, Germany) and corneal topography with Orbscan II (Bausch & Lomb, Rochester, NY, USA). Postoperative evaluation was done at least 1 month after cataract surgery. In G2, preoperative and postoperative endothelial cell densities were evaluated with noncontact specular microscope (Konan Medical, Inc., Hyogo, Japan).

Refractive and corneal astigmatisms were converted to vector notations by using the following equations by Fourier analysis [16]: J0 = −(C/2)*cos2θ, J45 = −(C/2)*sin2θ (C = minus cylinder power of refractive errors, θ = cylinder axis of refractive errors, J0 < 0 against the rule astigmatism, J0 > 0 with the rule astigmatism, J45 accounts for oblique astigmatism), J0 and J45 were converted into Cartesian coordinates as J0 being the x axis and J45 being the y axis. The value (0, 0) represents an eye free of astigmatism.

The mean numerical error (MNE) was defined as the arithmetic mean of the prediction errors calculated by subtracting the postoperative SE from preoperatively intended SE. MNE lower than zero means a hyperopic shift from intended SE and higher than zero means a myopic shift from intended SE. The mean absolute error (MAE) was defined as the mean of magnitude of prediction errors and was calculated to analyze each toric IOL’s target accuracy.

### IOL calculations

To determine IOL cylinder power, calculation was done using each IOL manufacturer’s provided online toric IOL calculators. Cylinder power of Acrysof toric (Alcon Laboratories, Inc.) was calculated through Alcon Online Toric IOL Calculator website (https://www.myalcon-toriccalc.com). The required parameters were entered as follows; axial length, anterior chamber depth, flat and steep K, and flat and steep meridian were provided by IOL Master 500 (Carl Zeiss Meditec AG, Jena, Germany), surgically induced astigmatism was entered as zero, incision location was temporal (either 180 or 0 degree according to laterality). Cylinder power of Tecnis toric (Abbott Medical Optics, Inc.) was calculated through Tecnis Toric Aspheric IOL website (https://www.amoeasy.com/calc). The required parameters were entered as follows; axial length, flat and steep K, and flat and steep meridian were provided by IOL Master 500 (Carl Zeiss Meditec AG, Jena, Germany), surgically induced astigmatism was entered as zero, incision location was temporal (either 180 or 0 degree according to laterality), A-constant 119.30 and K-constant 1.3375. Cylinder power of Zeiss toric (Carl Zeiss Meditec AG) was calculated through Z CALC Online IOL Calculator website (https://zcalc.meditec.zeiss.com). The required parameters were entered as follows; axial length, anterior chamber depth and keratometry (flat and steep K, and their meridians) were provided by IOL Master 500 (Carl Zeiss Meditec AG, Jena, Germany), surgically induced astigmatism was entered as zero, incision orientation was temporal (either 180 or 0 degree according to laterality).

Target SE power of implanted IOL was calculated preoperatively according to surgeon and patients’ agreed target. For subjects who received implantation of Acrysof toric (Alcon Laboratories, Inc.) or Tecnis toric (Abbott Medical Optics, Inc.), target SE power of IOL was calculated using SRK/T formula. For subjects who received implantation of Zeiss toric (AT TORBI 709 M, Carl Zeiss Meditec AG), target SE power of IOL was calculated and provided from Z CALC Online IOL Calculator website (https://zcalc.meditec.zeiss.com).

### Surgical technique

One experienced ophthalmology surgeon (M. K. Kim) performed all surgeries under topical and subtenon anesthesia. Before surgery, 0 and 180 axis marking by corneal dimpling with Sinskey hook (Katena, Denville, NJ, USA) was done in all subjects seating upright at slit-lamp using horizontal slit beam. Intraoperative marking with Mendez ring (Katena) at implantation meridian was done. Continuous curvilinear capsulorrhexis and coaxial phacoemulsification was performed through a 2.7-mm temporal corneal incision. Toric IOL was inserted into the capsular bag using injector and disposable cartridge system. IOL was rotated to align IOL cylinder axis with the steepest corneal axis. After removal of viscoelastics, IOL cylinder axis was evaluated and repositioned to meet targeted position. Finally, balanced salt solution (Alcon, Fort Worth, TX, USA) as irrigating solution was injected into incision site to close corneal incision causing edema. After surgery, postoperative eye drops of antibiotics, corticosteroids, and nonsteroidal anti-inflammatory eye drops were prescribed to all patients.

### Statistical analysis

Statistical analysis was performed using the SPSS software for Windows version 22.0 (SPSS, Inc., Chicago, IL). Two tailed paired and unpaired t-test were used to assess preoperative and postoperative differences. Analysis of variance test was used to evaluate inter-group differences. A probability value of *p* < 0.05 was considered statistically significant. The results are presented as mean ± standard error of mean (SEM) unless otherwise indicated.

## Results

A total of 370 eyes were evaluated. Average preoperative BCVA were 0.526 ± 0.029 logMAR and 0.571 ± 0.110 logMAR for G1 and G2, respectively. Preoperative minus cylinder power of corneal astigmatism was − 2.141 ± 0.047 D and − 4.030 ± 0.438 D for G1 and G2, respectively. Average evaluation time after cataract surgery was 1.77 and 2.52 months for G1 and G2, respectively. Average interval time between PKP and cataract surgery was 42.33 months in G2 (not shown in Table 1). In G2, average skewed radial axes was 26.67 degrees and showed no significant difference among all types of IOL implanted (*p* = 0.976, not shown in Table 1). Demographics and characteristics of each group are shown in Table 1.

**Table 1:** Demographics and clinical characteristics of subjects with normal corneal astigmatism (G1) or post-PKP astigmatism (G2)

	G1	G2
**Male**: **Female**	125 (35.8%): 224 (64.2%)	13 (61.9%): 8 (38.1%)
**Age (years)**	62.06 ± 0.885	50.00 ± 2.540
**DM**	65 (18.6%)	2 (9.5%)
**Implanted IOL type**		
**Front-Toric IOL**	85 (24.4%)	2 (9.5%)
**Back-Toric IOL**	174 (49.9%)	13 (61.9%)
**Bi-Toric IOL**	90 (25.8%)	6 (28.6%)
**Preoperative BCVA (logMAR)**	0.53 ± 0.30	0.57 ± 0.11
**Preoperative Minus Cylinder Power of Corneal Astigmatism (D)**	−2.14 ± 0.47	−4.03 ± 0.43
**Preoperative Minus Cylinder Power of Refractive Astigmatism (D)**	−2.56 ± 0.09	−4.95 ± 0.47
**Evaluation Time After Cataract Surgery (months)**	1.77 ± 0.08	2.52 ± 0.42

*G1* normal corneal astigmatism group, *G2* post-penetrating keratoplasty astigmatism group, *DM* diabetes mellitus, *IOL* intraocular lens, *BCVA* best corrected visual acuity, *D* diopters

### Refraction errors and astigmatism vector analysis

**Fig. 1:**
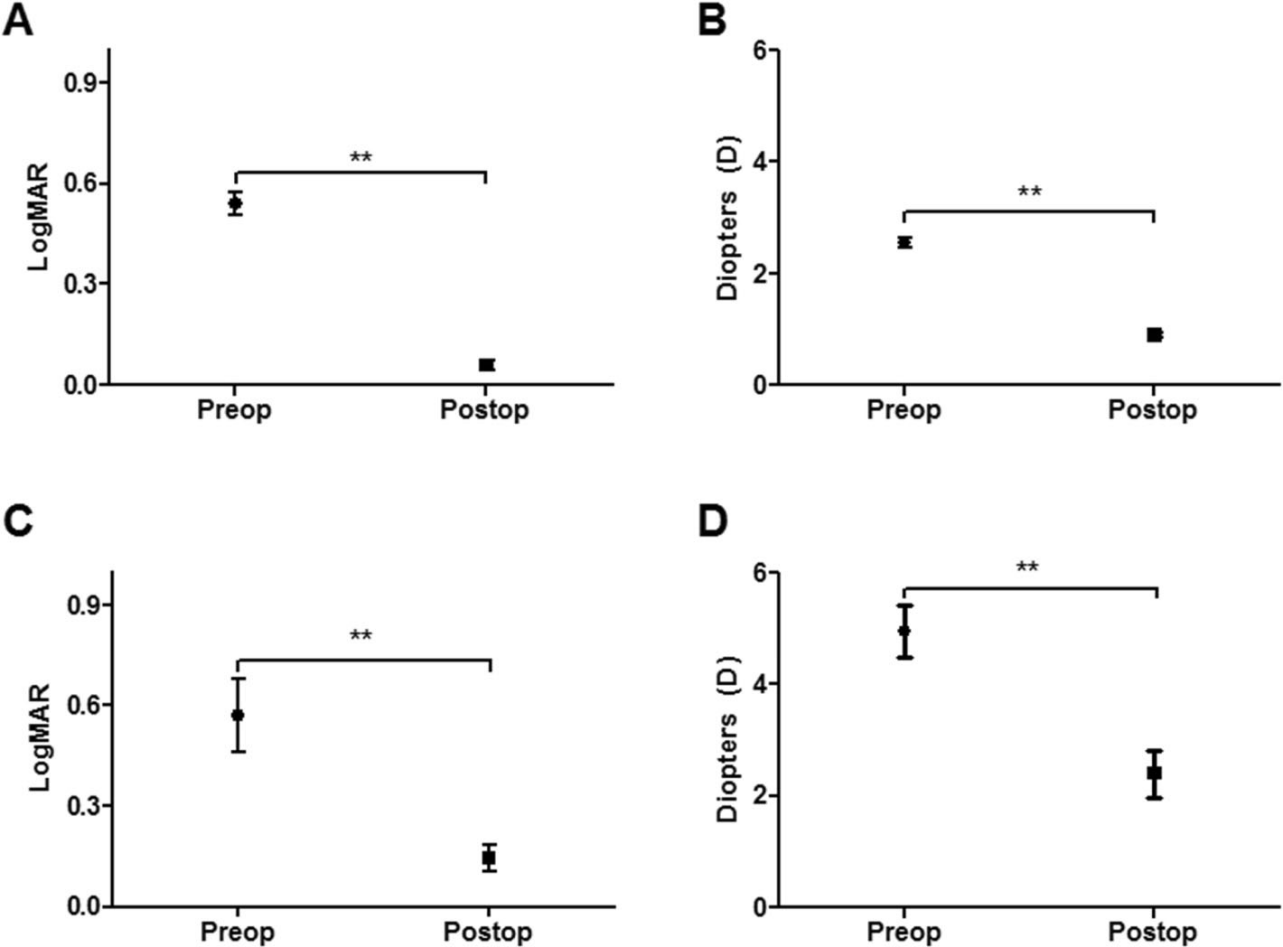
Preoperative and postoperative BCVA and minus cylinder power of refractive astigmatism in G1 and G2. Significant improvement in BCVA was observed in both groups (**a**: G1, **c**: G2) (**p < 0.001, paired t-test). Significant reduction in minus cylinder power of refractive astigmatism was observed in both groups (**b**: G1, **d**: G2) (**p < 0.001, paired t-test). G1 = normal corneal astigmatism group, G2 = post-penetrating keratoplasty astigmatism group, BCVA = best corrected visual acuity

**Fig. 2:**
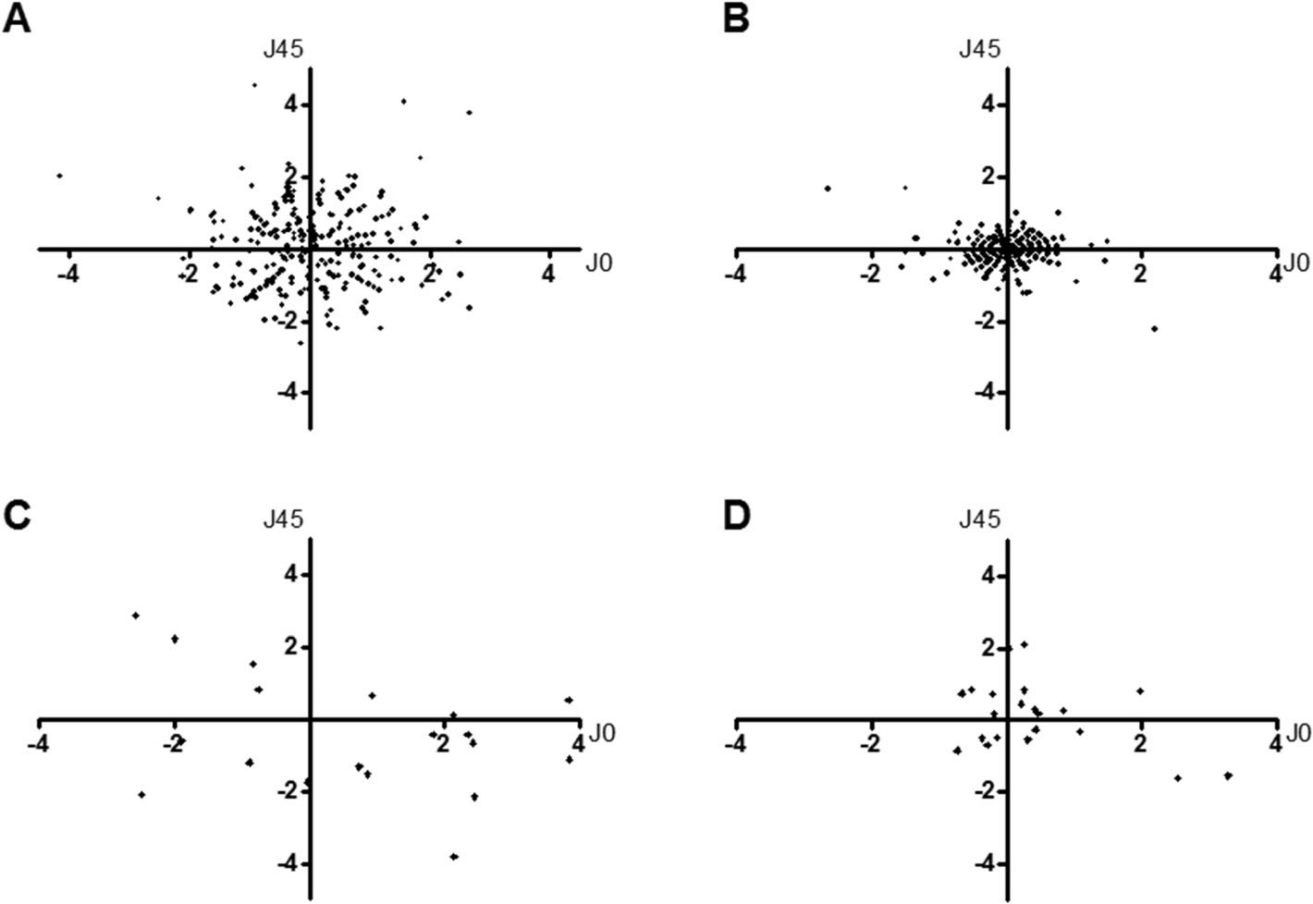
Vector analysis of refractive astigmatism in G1 and G2.. Refractive astigmatism was converted to vector notations by using equations by Fourier analysis (J0 = −(C/2)*cos2θ, J45 = −(C/2)*sin2θ (C = minus cylinder power of refractive errors, θ = cylinder axis of refractive errors, J0 < 0 against the rule astigmatism, J0 > 0 with the rule astigmatism, J45 accounts for oblique astigmatism), x axis = J0, y axis = J45). In both groups, clustering of dots toward (0, 0), postoperatively (**b**: G1, **d**: G2) compared to preoperative J0 / J45 (**a**: G1, **c**: G2) was observed. G1 = normal corneal astigmatism group, G2 = post-penetrating keratoplasty astigmatism group

We evaluated the clinical effect on reduction of astigmatism in either normal or post-PKP cornea. In G1 and G2, postoperative improvement of BCVA and decrease in cylinder power of manifest refraction error were significant (*p* < 0.001 in both groups, Fig. 1). Both group’s vector analysis of manifest refractive error astigmatism converted into Cartesian plots revealed clustering of plots toward (0, 0), postoperatively (Fig. 2).

**Fig. 3:**
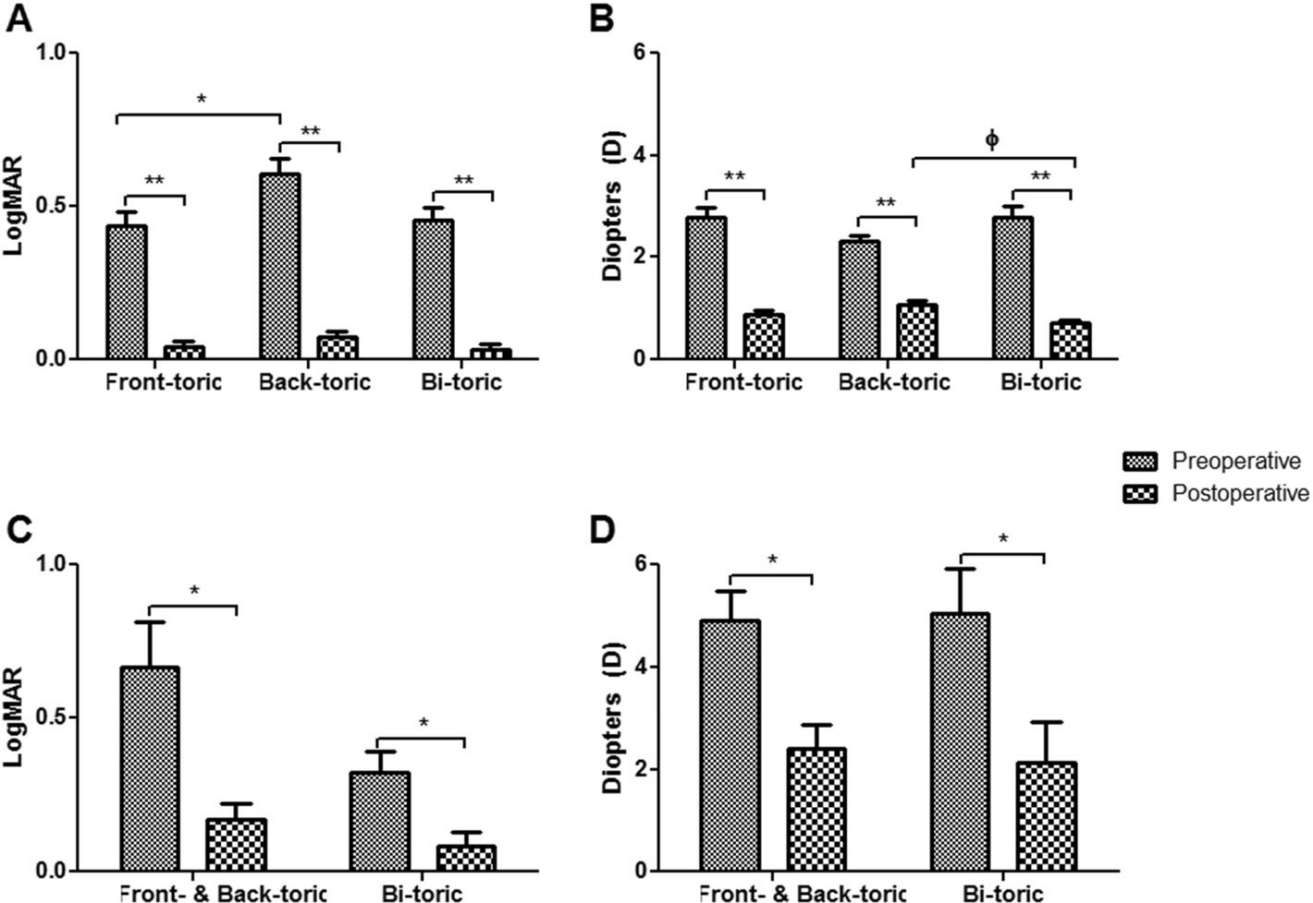
Subgroup analysis of BCVA and minus cylinder power of refractive astigmatism according to type of IOL in each group. The minus cylinder power of refractive astigmatism was converted to absolute numerical value for easier understanding. Significant improvement in postoperative BCVA was observed in all types of toric IOLs and no difference was seen among all types of toric IOL, in both groups (**a, c**) (**p = < 0.001 in G1, *p < 0.05 in G2, paired t-test). There was preoperative difference in BCVA between front- and back-toric IOL in G1 (A) (*p < 0.05, unpaired t-test), while there was no difference among all types of IOL in G2 (C) (*p* > 0.05, unpaired t-test). Significant improvement in postoperative minus cylinder power of refractive astigmatism was observed in all types of toric IOLs in both groups (**b, d**) (**p = < 0.001 in G1, *p < 0.05 in G2, paired t-test). Bi-toric IOL showed significantly better postoperative minus cylinder power of refractive astigmatism compared to back-toric IOL in G1 (B) (Φp = 0.001, unpaired t-test), while there was no difference in G2 (D) (p > 0.05, unpaired t-test). G1 = normal corneal astigmatism group, G2 = post-penetrating keratoplasty astigmatism group, BCVA = best corrected visual acuity, IOL = intraocular lens

**Fig. 4:**
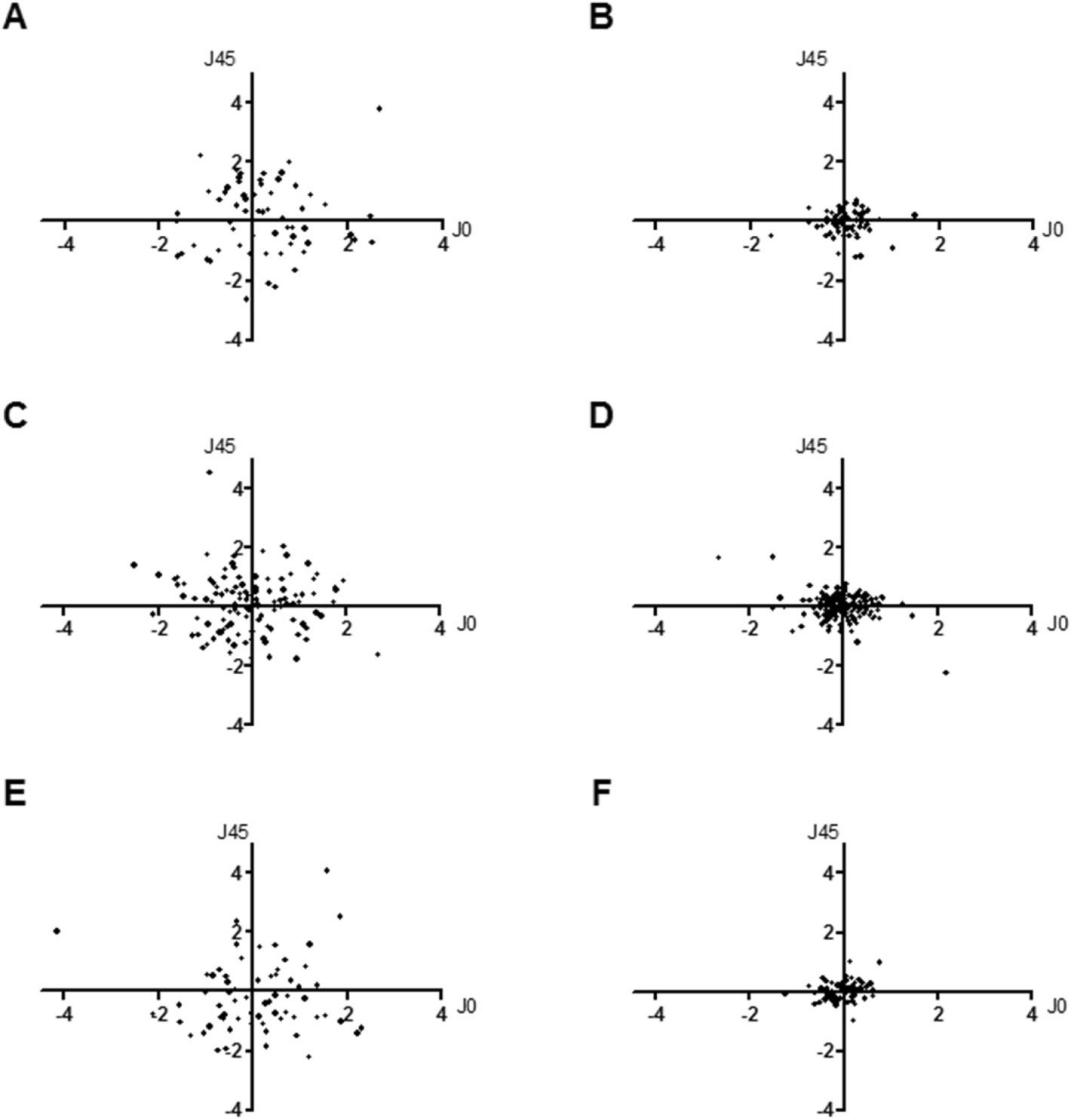
Sub-group vector analysis of refractive astigmatism according to type of IOL in G1. Refractive astigmatism was converted to vector notations by using equations by Fourier analysis (J0 = −(C/2)*cos2θ, J45 = −(C/2)*sin2θ (C = minus cylinder power of refractive errors, θ = cylinder axis of refractive errors, J0 < 0 against the rule astigmatism, J0 > 0 with the rule astigmatism, J45 accounts for oblique astigmatism), x axis = J0, y axis = J45). All types of toric IOLs showed clustering of dots toward (0, 0), postoperatively. Front-toric IOL: A = preoperative, B = postoperative, Back-toric IOL: C = preoperative, D = postoperative, Bi-toric: E = preoperative, F = postoperative. IOL = intraocular lens, G1 = normal corneal astigmatism group

Sub-group analysis was conducted in each group depending on the type of toric IOL. Aside from a preoperative difference in BCVA between front- and back-toric IOL in G1 (*p* = 0.028, Fig. 3), there was no other preoperative difference among type of IOL in G1 and G2 (*p* > 0.05 in both groups, Fig. 3). In both groups, all types of toric IOL significantly improved postoperative BCVA and reduced cylinder power of refractive astigmatism (*p* < 0.001 and *p* < 0.05, respectively, Fig. 3). No significant difference in BCVA depending on types of toric IOL was observed (p > 0.05 in both groups, Fig. 3). In G1, bi-toric IOL showed significantly better postoperative cylinder power of manifest refraction errors compared to back-toric IOL (*p* = 0.001, Fig. 3), while there were no differences between front- and back-toric IOLs, and front- and bi-toric IOLs (*p* = 0.124 and *p* = 0.491, respectively, Fig. 3). In G2, there was no significant difference in postoperative cylinder power of manifest refraction errors between types of toric IOL (*p* = 0.657, Fig. 3). Also refractive astigmatism vector analysis according to the type of IOL implanted revealed all types to show postoperative clustering toward (0,0) (Fig. 4).

### Mean numerical error and mean absolute error

**Fig. 5:**
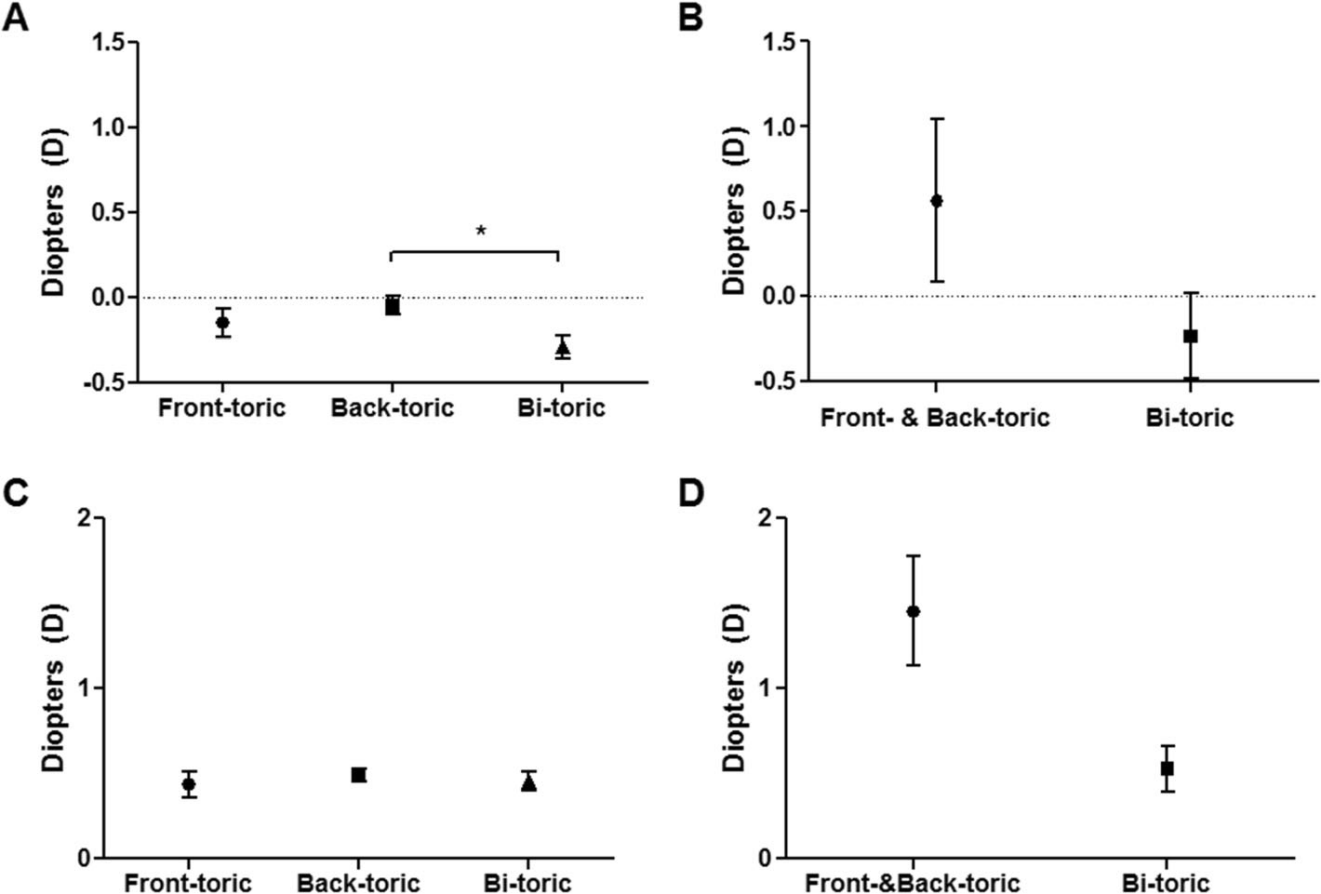
Mean numerical and absolute errors according to type of IOL in each group. Upper level graphs refer to mean numerical error and lower level graphs refer to mean absolute error. A and C are G1, while B and D are G2. Bi-toric IOL showed significantly more hyperopic shift of 0.291 ± 0.616 D compared to back-toric IOL (0.045 ± 0.053 D) in G1 (*p = < 0.05, ANOVA) (**a**), while there was no difference in G2 (**b**). No significant difference in mean absolute error in both groups was observed according to type of IOL (**c, d**). IOL = intraocular lens, D = diopters, G1 = normal corneal astigmatism group, G2 = post-penetrating keratoplasty astigmatism group, ANOVA = Analysis of variance test

We analyzed target accuracy of postoperative SE depending on the type of toric IOL. In G1, MNE revealed bi-toric IOL to show a more significant hyperopic shift of 0.291 ± 0.616 D compared to back-toric IOL (*p* = 0.021, Fig. 5). While, in G2, there was no significant difference in MNE among the types of IOL (*p* = 0.322, Fig. 5). In both G1 and G2, there was no significant difference in MAE according to type of IOL (*p* = 1.000 and *p* = 0.091, respectively, Fig. 5).

### Complications

**Fig. 6:**
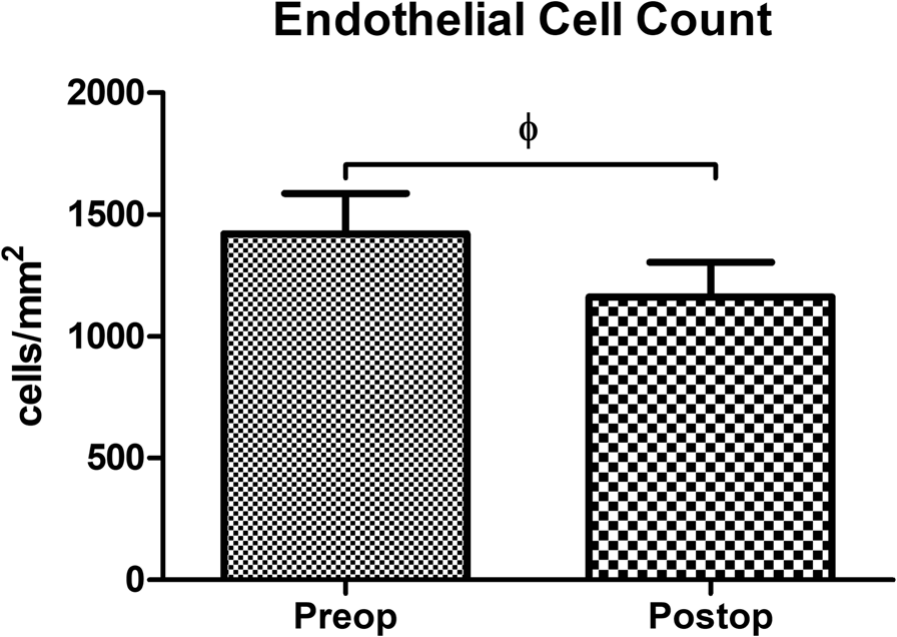
Endothelial cell count in post-PKP group (G2). Endothelial cell density was significantly reduce, postoperatively (Φp = 0.001, paired t-test). G2 = post-penetrating keratoplasty astigmatism group

In both G1 and G2, no intraoperative and postoperative complications that acquired additional interventions were noted. Although, in G2, significant reduction in endothelial cell density postoperatively was observed (*p* = 0.001, Fig. 6), it did not show any clinically relevant corneal edema nor graft failure.

## Discussion

This study showed that all types of toric IOLs can significantly reduce astigmatism and enhance visual acuity in both normal and post-PKP corneal astigmatism. This was confirmed with vector analysis converted to Cartesian coordinates showing postoperative dots clustering toward (0, 0). These results correlate with previous studies reporting the beneficial effects of various toric IOLs for not only normal corneal astigmatism but also other types of corneal astigmatisms caused by either an underlying corneal disease or postoperative complication [4, 6, 10, 17].

To date, most studies regarding toric IOLs have been focused on a single type of toric IOL. Also, recent comparative studies have only reported on the postoperative influences according to IOL haptic design or the effects between only two types of commercially manufactured toric IOLs [4, 13, 14, 18]. To the best of our knowledge, this is the first study to compare the effects of astigmatism correction according to type of IOL; front-, back- and bi-toric IOLs.

Ferreira and Almeida reported that there was no significant difference between front- and back-toric IOL concerning refractive results [13]. Seth et al. reported that there was no difference in refractive results between back- and bi-toric IOLs [19]. Also Scialdone et al. showed similar results of no statistical difference in refractive astigmatism between back- and bi-toric IOLs [20]. However, in this study, though there was no difference in the resulting refractive astigmatism between front- and back-toric IOLs in normal corneal astigmatism, bi-toric IOL had shown to be more superior to back-toric IOL. The correction efficacy may be affected by capability of the astigmatic correction power originally incorporated in IOL design, stability of the axis after implantation, the accuracy of each toric IOL power program, and consideration of posterior corneal astigmatism etc. [21]. Despite the better outcome of bi-toric IOL in normal corneal astigmatism, there was no significant difference in post-PKP corneal astigmatism. High skewed radial axes and irregular astigmatism may have contributed to lowering the effect of toric IOL on post-PKP astigmatism.

There was no difference in MAE among all types of toric IOLs in both normal and post-PKP corneal astigmatism groups. In other words, our study showed that the accuracy of all types of toric IOLs did not differ using SRK/T formula for front-and back-toric IOL, and the provided Z CALC Online IOL Calculator for bi-toric IOL. Recently, Eom et al. reported that Haigis formula was more accurate in predicting refractive outcomes than SRK/T formula [21]. However, this result was confined to back-toric IOL only and therefore, further studies concerning IOL power calculating formulas and their use according to type of IOL may be needed.

While the post-PKP corneal astigmatism group showed no difference in MNE among types of IOL, the bi-toric IOL in normal corneal astigmatism group showed the most hyperopic shift of about 0.291 ± 0.066 D which was significant compared to back-toric IOL (MNE was average 0.045 ± 0.053 D, *p* = 0.021) but not front-toric IOLs (MNE was average − 0.140 ± 0.081 D, *p* = 0.902). Similar to our results, Scialdone et al. reported that back-toric IOL was significantly nearer to emmetropia compared to bi-toric IOL [20]. Therefore, hyperopic shift tendency should be considered when implanting bi-toric IOLs.

There were no postoperative complications that needed secondary interventions in all groups in this study. Though, in the post-PKP group, there was significant reduction in endothelial cell count that was not clinically significant.

There are some limitations to this study. Due to the retrospective nature of this study, the evaluation of IOL rotation was not able to be retrieved. Recent studies have reported up to 3.3% of cylinder power correction loss per degree of IOL rotation and that misalignment of greater than 30 degrees loses all effect of astigmatism correction of toric IOLs [22]. Therefore IOL rotation evaluation is crucial. However, several studies have proven that most commercially manufactured toric IOLs have excellent rotational stability [7, 13, 18, 23, 24]. Therefore, most of the subjects included in our study would also have had a high possibility of excellent rotational stability. Also, since this was a retrospective study, postoperative follow up periods were not consistent among subjects. At our cornea and cataract clinic, postoperative follow up is usually on the next day, at 1 week and 1 month after surgery, but some subjects might have missed their appointments or performed their postoperative evaluation on another day or had wanted a longer follow up period. For these reasons, our study subjects had different postoperative evaluation time, which might have affected the results. However, follow up periods among subjects did not differ largely and it is known that there is not much change in refraction after 1 month of cataract surgery [5, 10, 20]. Still, a future study with consistent postoperative evaluation time would be beneficial to accurately evaluate toric IOL’s effects. In addition, because of our clinic’s routine follow up protocol after cataract surgery, postoperative evaluation time was only performed approximately at one to 2 months after surgery. However, according to past studies, toric IOLs have shown stable refraction with minimal IOL rotation after 1 month from surgery and no significant differences were seen when compared to 3 and 6 months after surgery [5, 10, 20]. Therefore, despite our relatively short study period, our study’s current results may not differ significantly from long term outcomes, if they had been performed. Another limitation is that though this study focused on the types of toric IOLs by the location of cylinder power, we only included one commercially manufactured IOL in each subgroup. Therefore, each IOL used in this study might not sufficiently represent each subgroup. However, the commercially manufactured toric IOLs included in our study are one of the most world-widely used toric IOLs, which make the comparative results from this study meaningful. Also, the number of subjects who had implantation of front-toric IOL in G2 was only two, which may not be enough to properly represent front-toric IOL used in post-PKP subjects. For this reason, we had combined front- and back-toric IOL as one group to compare them to bi-toric IOL. Therefore, further investigation with a larger study group to identify front-toric IOL’s sole effect in post-PKP subjects is necessary. Lastly, the overall number of subjects included in the post-PKP group was much smaller than the normal corneal astigmatism group. This was most likely due to the exclusion criteria, where subjects with ocular diseases that may affect vision or astigmatism were excluded.

## Conclusions

In conclusion, our study shows that implantation of bi-toric, front-toric, and back-toric IOLs are all beneficial and comparably effective in correcting both normal and post-PKP corneal astigmatisms. Also, bi-toric IOL shows better results in refractive astigmatism and more postoperative hyperopic SE compared to back-toric IOLs in normal corneal astigmatism. While in post-PKP corneas, all types of toric IOLs showed similar results.

## Data Availability

The datasets used and/or analyzed during the current study are available from the corresponding author on reasonable request.

## Abbreviations

BCVA: Best corrected distance visual acuity
D: Diopters
DM: Diabetes mellitus
G1: normal corneal astigmatism group
G2: post-penetrating keratoplasty astigmatism group
IOL: Intraocular lens
MAE: Mean absolute error
MNE: Mean numerical error
PKP: Penetrating keratoplasty
SE: Spherical equivalent
SEM: Standard error of mean
UCVA: Uncorrected distance visual acuity
